# High compassion predicts fewer sleep difficulties: A general population study with an 11‐year follow‐up

**DOI:** 10.1002/brb3.3165

**Published:** 2023-08-22

**Authors:** Iina Tolonen, Aino Saarinen, Sampsa Puttonen, Mika Kähönen, Mirka Hintsanen

**Affiliations:** ^1^ Division of Psychology, Faculty of Education and Psychology University of Oulu Oulu Finland; ^2^ Department of Psychology and Logopedics, Faculty of Medicine University of Helsinki Helsinki Finland; ^3^ Faculty of Social Sciences Tampere University Tampere Finland; ^4^ Finnish Institute of Occupational Health Helsinki Finland; ^5^ Department of Clinical Physiology, Tampere University Hospital and Faculty of Medicine and Health Technology Tampere University Tampere Finland

**Keywords:** compassion, insomnia, longitudinal, personality, sleep

## Abstract

**Introduction:**

This study investigated the cross‐sectional and longitudinal associations between self‐reported compassion and sleep quality.

**Methods:**

The data came from the population‐based Young Finns Study with an 11‐year follow‐up on compassion and sleep (*n* = 1064). We used regression models, multilevel models, and cross‐lagged panel models to analyze the data.

**Results:**

The results showed that high compassion was cross‐sectionally associated with lesser sleep deficiency and fewer sleep difficulties. High compassion also predicted fewer sleep difficulties over an 11‐year follow‐up (adjusted for age, gender, socioeconomic position in childhood and adulthood, body mass index, health behaviors, and working conditions). This association disappeared when controlling for depressive symptoms. The predictive pathway seemed to proceed more likely from high compassion to fewer sleep difficulties than vice versa.

**Discussion:**

Compassion may buffer against sleep difficulties, possibly via reducing depressive symptoms.

## Graphical abstract

Compassion is associated with better sleep and can protect against sleep difficulties. The protective effect of compassion may emerge due to fewer depressive symptoms.

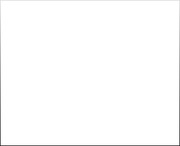

## 1 INTRODUCTION

Difficulties with sleep have become one of the leading health issues in Western countries (Chattu et al., 2019). Sleep difficulties can refer to problems in falling asleep, staying asleep, feeling tired after a night's sleep (Jenkins et al., 1988), or perceiving sleep deficiency or having an excessive amount of sleep (Hublin et al., 2007; Johnson & Czeisler, 2022; Kronholm et al., 2011). It has been estimated that as much as 23%–56% of the general population have intermitted difficulties with sleep (Kronholm et al., 2016; Léger et al., 2008).

Sleep difficulties are problematic as they are linked with a variety of negative outcomes. A great amount of evidence has shown that sleep difficulties are associated with, for example, impaired work ability in terms of increased workplace accidents and increased sick leaves (Amiri & Behnezhad, 2020; Uehli et al., 2014). Especially if they become chronic, perceived sleep deficiency and sleep difficulties can increase the risk for a variety of somatic diseases and psychiatric disorders, such as depression (Bao et al., 2017; Pandi‐Perumal et al., 2020), metabolic syndrome (Altman et al., 2012; Jennings et al., 2007), and cardiovascular‐related mortality (Kronholm et al., 2011; Shankar et al., 2010; Sofi et al., 2014).

To reduce sleep difficulties, it is necessary to increase the understanding of their etiology. To date, well‐known risk factors for sleep difficulties include, for example, perceived stress, pain, depression, and physical illnesses (Kamdar et al., 2012; Roehrs & Roth, 2005; Sanford et al., 2015; Zhai et al., 2015), adverse health behaviors and irregular work conditions (Kredlow et al., 2015; Pasman et al., 2020; Rahim et al., 2021), and anxiety‐prone personality traits such as high neuroticism and type D personality (Hintsanen, 2014; Juskiene et al., 2018; Stephan et al., 2018).

The study of positive dispositional traits with sleep has been sparser, despite the accumulating evidence of their association with mental and physical well‐being (Allen & Leary, 2010; Saarinen, Keltikangas‐Järvinen, Hintsa, et al., 2020; Solberg Nes & Segerstrom, 2006). One unexplored disposition, which could be associated with sleep, is compassion. Compassion refers to one's disposition to experience concern for others’ suffering and a desire to alleviate it (Goetz et al., 2010). To date, no cross‐sectional or longitudinal study has examined whether compassion for others could protect against sleep difficulties.

Although studies on compassion for others and sleep are entirely lacking, there are some single and inconclusive findings on the association of compassion‐related traits with sleep. Some cross‐sectional studies have found that a greater tendency to be warm and sympathetic is associated with, for example, lesser perceived sleep deficiency (Cellini et al., 2017; Hintsanen, 2014; Randler, 2008), whereas other studies have resulted in null findings (Stephan et al., 2018; Sutin et al., 2020). Additionally, prosocial behaviors, a close construct to compassion (Goetz et al., 2010) have been reported to correlate with lesser sleep difficulties in a cross‐sectional study (Melton et al., 2021).

Past research suggests that compassion could effectively protect against sleep difficulties via several mechanisms. First, compassionate individuals report lower stress levels, have lower stress‐reactivity, and have higher stress‐buffering factors such as higher perceptions of social support (Abelson et al., 2014; Brito‐Pons et al., 2018; Cosley et al., 2010). Second, high compassion is clearly associated with components of effective emotion regulation, including more frequent use of acceptance and upward regulation of positive affect (Engen & Singer, 2014; Jazaieri et al., 2018). There is strong evidence that both lower stress and adaptive emotion regulation strategies are, in turn, linked with fewer sleep difficulties (de Grey et al., 2018; Ong et al., 2012; Sanford et al., 2015).

Reverse associations from frequent sleep difficulties to lowered levels of compassion are also plausible. Specifically, there is evidence that sleep difficulties can impair certain socio‐cognitive skills, such as social decision‐making and prosocial behaviors (Dickinson & McElroy, 2017; Holbein et al., 2019; Satterfield et al., 2019) that are, in turn, essential parts of other‐directed compassion (Goetz et al., 2010). Following this, a prospective investigation found that a greater baseline in sleep difficulties was associated with a decrease in the disposition to be warm and sympathetic (Stephan et al., 2018). No study has investigated, however, whether the predictive pathway is more likely to proceed from compassion to sleep difficulties or vice versa.

The current study aims to examine the relationship of compassion with a variety of sleep indicators. More specifically, we aim to investigate (1) whether compassion is cross‐sectionally associated with sleep quantity and quality (i.e., sleep duration, perceived sleep deficiency, and sleep difficulties), (2) whether compassion predicts the trajectory of sleep difficulties over an 11‐year follow‐up, and (3) whether a possible predictive relationship is more likely to proceed from compassion to sleep difficulties, or vice versa. We also aim to tentatively test, if depressive symptoms may be the mediator behind the association of compassion and sleep. The participants came from the prospective Young Finns Study (YFS) (Raitakari et al., 2008) including an 11‐year follow‐up of compassion and sleep difficulties. We were able to take into consideration many possible confounders, such as body mass index (BMI) (Taheri et al., 2004), health behaviors (Kredlow et al., 2015; Pasman et al., 2020), working conditions (Mai et al., 2019; Rahim et al., 2021), and depressive symptoms (Bao et al., 2017; Zhai et al., 2015).

## 2 METHODS

### 2.1 Participants

The participants came from the ongoing, prospective YFS. The baseline measurement of YFS was conducted in 1980. Since then, participants have been followed up for 37 years on several occasions (in 1983, 1986, 1989, 1992, 1997, 2001, 2007, 2012, and 2017). In the baseline measurement of 1980, the sample size was 3596. The sample consisted of 6 age cohorts, aged between 3 and 18 years at the baseline (born in 1962, 1965, 1968, 1971, 1974, or 1977). The sampling was designed to collect a population‐based sample, meaning noninstitutionalized Finnish children who were representative regarding sex (male vs. female), urban versus rural areas, and Eastern versus Western regions of Finland.

The five Finnish universities with medical schools collaborated on the sampling (i.e., the Universities of Helsinki, Turku, Tampere, Oulu, and Kuopio). More specifically, half of the subjects were invited from the university cities and the other half from their rural municipalities. The rural municipalities were selected on the basis that they (1) were within 200 km distance from the respective university city, (2) had an approximately similar industrial structure to each other, and (3) had a large enough population of children belonging to the target age cohorts. In practice, participants were selected from the five university cities, and two rural municipalities from the regions of Helsinki, Turku, and Tampere (i.e., West). Two municipalities were selected from the region of Oulu and four rural municipalities were selected from the region of Kuopio (i.e., East).

Thereafter, the individuals born in 1962, 1965, 1968, 1971, 1974, or 1977, living in each target city and rural municipality, were retrieved from the population register of the Social Insurance Institution and put in random order. Next, the researchers invited 30 individuals from each sampling group (i.e., a certain age cohort, sex group, and city/municipality, e.g., boys born in 1962 in Turku). Altogether 4320 individuals were invited, and 3596 of them participated in the baseline study. The design and progression of the YFS are described with further details elsewhere (e.g., Åkerblom et al., 1985; Raitakari et al., 2008).

The YFS has been carried out under the Declaration of Helsinki, and the study design has been approved by the ethical committees of all Finnish Universities with a medical faculty (Universities of Helsinki, Turku, Tampere, Kuopio, and Oulu). All the participants or their parents (if participants were aged <18 years) provided informed consent before participation.

In this study, we included all the participants who had data available on compassion (2001 and 2012) and sleep indicators (2001, 2007, and 2012) in at least one of the measurements years and who had full data on the covariates (including age, gender, childhood and adulthood socioeconomic position [SEP] in 1980 and 2011, health behaviors in 2011, working conditions in 2011, and depressive symptoms in 2012). Since pregnancy is associated with increased sleep difficulties, pregnant participants and those who did not provide information about possible pregnancy were excluded (*n* = 9; (Sedov et al., 2018). Hence, the final sample size was 1064.

### 2.2 Measurements

#### 2.2.1 Dispositional compassion

Dispositional compassion was measured using the subscale of Compassion (vs. revengefulness) from the Temperament and Character Inventory (Cloninger et al., 1994). The subscale consists of 10 items such as “I hate to see anyone suffer” or “It gives me pleasure to help others, even if they have treated me badly.” All the items were responded to with a five‐point scale (1 = Completely disagree; 5 = Completely agree). The scale had high internal consistency (Cronbach's *α* = .87 in 2001, *α* = .85 in 2012) and high test–retest correlation between follow‐up measurements (*r* = .70). The mean score of the scale was calculated for all the participants who had responded to at least 50% of the items. Construct validity and reliability of the compassion scale have been described in more detail elsewhere (Hintsanen et al., 2019).

#### 2.2.2 Sleep

Indicators of sleep included sleep duration, perceived sleep deficiency, sleep problems, and sleep difficulties. We used the term sleep problems for Jenkins's Sleep Scale (Jenkins et al., 1988) and the term sleep difficulties for sleep items from the Maastricht Vital Exhaustion Questionnaire (VEQ) (Appels et al., 1987) to differentiate these two measures from each other.

Sleep duration was reported as habitual hours of sleep per night. Sleep duration was classified into three categories (1 = Sleep duration of 6.5 h or less per night; 2 = Sleep duration between 7 and 8.5 h per night (reference group); 3 = Sleep duration of 9 h or more per night). Past research has used similar groupings (Cappuccio et al., 2010).

Perceived sleep deficiency was calculated with the following formula: perceived sleep deficiency = perceived need for sleep in hours—reported hours of sleep per night. Thus, greater scores indicated greater perceived sleep deficiency. Perceived need for sleep and hours of sleep per night were assessed in 2012 by asking the participants to select the appropriate length from 10 options (i.e., 5 h or less, 6, 6.5, 7, 7.5, 8, 8.5, 9, 9.5, or 10 h or more).

Sleep problems were assessed in 2012 using Jenkins's Sleep Scale, which aims to capture the frequency of most reported sleep symptoms (Jenkins et al., 1988). The scale is made of four items measuring difficulties in falling asleep, difficulties remaining asleep, waking up several times during a night, and feeling tired after a usual amount of sleep at night (in the past 4 weeks). The items were rated with a 6‐point scale (1 = Never, 2 = 1–3 nights/month, 3 = Approximately 1 night/week, 4 = 2–4 nights/week, 5 = 5–6 nights/week, 6 = Every night). The items are similar to the insomnia criteria as identified by the Diagnostic and Statistics Manual of Mental Disorders Fourth Edition (American Psychiatric Association, 1994). The scale has shown good internal consistency in the past (Juhola et al., 2021) and in the current study (Cronbach's *α* = .78). A sum score was calculated for the participants responding to all the items, meaning no missing values were allowed. Higher scores indicated greater sleep problems.

Sleep difficulties were measured with the sleep items of the Maastricht VEQ in 2001, 2007, and 2012 (Appels et al., 1987). We used the four sleep difficulty items of the questionnaire (“Do you often feel tired?”, “Do you often have trouble falling asleep?”, “Do you wake up repeatedly during the night?”, “Do you ever wake up with a feeling of exhaustion or fatigue?”). The items were responded with a 3‐point scale (0 = No, 1 = I don't know, 2 = Yes). High scores of the VEQ sleep items correlate with higher depressive symptoms and impaired health status (McGowan et al., 2004; Smith et al., 2009). Internal reliability of the scale was acceptable based on the inter‐item correlations (*r* = .18, *r* = .25, and *r* = .24 for 2001, 2007, and 2012, respectively) and the item‐scale correlations (*r* = .62–.73 in 2001, *r* = .67–.75 in 2007, and *r* = .65–.75 in 2012). For each measurement year, we calculated a sum score of VEQ sleep items, with higher scores indicating more severe sleep difficulties. As a sum value was calculated, no missing values were allowed.

#### 2.2.3 Covariates

Covariates included age, gender, childhood and adulthood SEP, working conditions, health behaviors, BMI, and depressive symptoms.

Childhood and adulthood SEP (including occupational status, educational level, and income) were assessed in 1980 and 2011, respectively. If the parental educational level or occupational status differed between the parents, the higher level or status was selected. The occupational status of the participants and their parents were coded into three categories (1 = Manual worker, 2 = Lower grade nonmanual worker, 3 = Upper grade nonmanual worker). The educational level of the participants and their parents included three categories (1 = Comprehensive school, 2 = High school or vocational school, 3 = Academic level, i.e., university or college). Childhood family income was reported with an 8‐point scale (1 = Less than 15,000 Finnish mark/year; 8 = More than 100,000 Finnish mark/year). Participants’ adulthood income was assessed with a 13‐point scale (1 = Less than 5000€/year; 13 = More than 60,000€/year). Each socioeconomic factor was entered as a separate variable in the analyses. Educational level and occupational status were treated as categorical variables and income as a continuous variable.

Working conditions (measured in 2011) included two variables. First, employment status was assessed with a dichotomous variable (unemployed vs. employed). Second, shift work within the past 12 months was classified into two categories (regular day work vs. shift work, i.e., morning/evening work, morning/evening/night work, evening/night shifts, irregular shifts).

Health behaviors (measured in 2011) included smoking, alcohol consumption, and physical activity. Smoking was coded as a dichotomous variable (daily smokers vs. others). Alcohol consumption was measured by the frequency of consuming six portions of alcohol at a time (one portion = 12 g; 1 = Twice a week or more, 2 = Once a week, 3 = 2–3 times a month, 4 = Once a month, 5 = 2–6 times a year, 6 = Rarely or never). Physical activity was assessed with a five‐item index (intensity, frequency, and duration of physical activity, and participation in structured sports activities). High scores on the physical activity index indicated higher physical activity. A more detailed description is available elsewhere (Telama et al., 1997). We also included BMI (kg/m^2^) and treated it as a continuous variable.

Depressive symptoms (measured in 2012) were assessed with Beck's Depression Inventory II (Beck et al., 1996). The scale consists of 21 items, and each response was selected from 4 alternatives (coded from 0 to 3), with higher scores indicating more severe depressive symptoms (*α* = .91). We calculated a sum score of the items for the participants who had responded to all items, meaning no missing values were allowed.

### 2.3 Statistical analyses

The data analysis was performed using STATA SE 16.1. Attrition analyses were carried out by comparing the included (*n* = 1064) and excluded/dropped‐out participants (*n* = 2532) with independent *t*‐tests and chi‐square tests. The statistical analyses with study variables and measurement years are summarized in Table 1.

**TABLE 1:** The study variables, measurement years, and employed statistical analyses.

	Measurement year			Statistical analysis		
The study variable	2001	2007	2012	Regression	Cross‐lagged panel model	Multilevel model
Compassion	x		x	x	x	x
Sleep duration			x	x		
Perceived sleep deficiency			x	x		
Sleep problems^a^			x	x		
Sleep difficulties^b^	x	x	x	x	x	x

Abbreviation: VEQ, Vital Exhaustion Questionnaire.

^a^ Sleep problems were measured with Jenkins's Sleep Scale.

^b^ Sleep difficulties were measured with sleep items from the VEQ.

First, we used regression analyses to investigate the cross‐sectional associations of dispositional compassion with indicators of sleep in 2012: perceived sleep deficiency, sleep problems, and sleep difficulties (linear regression analyses) and sleep duration (short vs. intermediate vs. long sleepers, multinomial regression analyses). We ran three models. Model 1 was adjusted for age and gender; Model 2 was adjusted also for SEP in childhood and adulthood, BMI, health behaviors (physical activity, smoking, and alcohol consumption), and working conditions (shift work, employment status); and Model 3 was adjusted for depressive symptoms in addition to the variables in Model 2.

Second, we investigated whether compassion predicts the trajectory of sleep difficulties (measured with the VEQ) over the 11‐year follow‐up (in 2001, 2007, and 2012). We used multilevel models with maximum likelihood estimation. We ran three models. In Model 1, fixed effects (interpreted as regression coefficients) were estimated for the intercept, compassion in 2001, age, age‐squared (to examine possible curvilinearity) and gender. Model 2 was also adjusted for SEP in childhood and adulthood, BMI, health behaviors, and working conditions; and Model 3 was further adjusted for depressive symptoms in addition to the variables in Model 2. In all the models, random effects included individual‐level variance of intercept and residual variance. In the analyses, age was centered to the age of the youngest age cohort in the first measurement year of sleep difficulties (i.e., to the age of 24 years), to reduce possible multicollinearity.

Third, we examined the predictive relationships between compassion and sleep difficulties (measured with the VEQ) in 2001 and 2012. We used cross‐lagged panel models, including four models for comparison: Model 1 (no predictive pathways between dispositional compassion and sleep difficulties, i.e., only autoregressive paths); Model 2 (a predictive pathway from compassion to sleep difficulties); Model 3 (a predictive pathway from sleep difficulties to compassion), and Model 4 (bidirectional predictive pathways). All the models included covariances between compassion and sleep difficulties within each measurement year. Further, all the models were adjusted for age, gender, and SEP in childhood and adulthood. We used four indices to compare the statistical fit of the models: root mean square error of approximation (RMSEA), comparative fit index (CFI), Bayesian information criterion (BIC) scores, and *χ* ^2^ test difference. The statistical fit of the model is estimated to be good when the values of RMSEA <.06 and the values of CFI > 0.95, whereas lower values of BIC and *χ* ^2^ indicate better statistical fit (Hu & Bentler, 1999; Schreiber et al., 2006).

## 3 RESULTS

The descriptive statistics of the study variables are presented in Table 2 and the bivariate correlations in Table 3. Attrition analyses demonstrated that there were no differences in compassion (2001), sleep duration (2012), or perceived sleep deficiency (2012) between included and dropped‐out participants. Moreover, there were no differences in participants’ or their parents’ occupational status or educational level between included and dropped‐out participants. Included participants had slightly higher income in adulthood (7.7 vs. 7.0, *p* < .001) and family income in childhood (4.9 vs. 4.8, *p* < .05). Women were more likely to participate than men (32.9% vs. 26.1%, *p* < .05), and included participants were slightly older than dropped‐out participants (32.0 vs. 31.2 years, *p* < .01). Included participants were slightly less likely to report sleep problems (9.3 vs. 9.9, *p* < .05 in 2012) and sleep difficulties (2.7 vs. 2.9, *p* < .05 in 2001) and had fewer depressive symptoms (4.4 vs. 6.1, *p* < .001) compared to dropped‐out participants.

**TABLE 2:** The means, standard deviations (SD), and frequencies of the study variables (*n* = 1064).

	Mean	*SD*	Range	*n*	%
Gender (female)				604	56.8
Age (2001)	32.0	5.0	24.0–39.0		
Parental educational level					
Comprehensive school				339	31.9
High school or occupational school				447	42.0
Academic level (university or college)				278	26.1
Parental occupational status					
Manual				395	37.1
Lower grade nonmanual				478	44.9
Upper grade nonmanual				191	18.0
Family income in childhood	4.9	1.9	1.0–8.0		
Participants’ educational level					
Comprehensive school				56	5.3
High school or occupational school				561	52.7
Academic level (university or college)				447	42.0
Participants’ occupational status					
Manual				552	51.9
Lower grade nonmanual				214	20.1
Upper grade nonmanual				298	28.0
Participants’ level of income	7.7	2.8	1.0–13.0		
Body mass index	26.4	4.9	17.0–58.5		
Physical activity	10.1	1.8	6.0–15.0		
Alcohol consumption	2.4	1.5	1–6		
Daily smokers				125	11.8
Shift work				312	29.3
Unemployed				92	8.7
Depressive symptoms	4.4	5.9	0.0–58.0		
Compassion (2001)	3.7	.6	1.0–5.0		
Sleep duration per night (2012)					
6.5 h or less				219	20.6
Between 7 and 8.5 h				801	75.3
9 h or more				22	4.1
Sleep deficiency (2012)	1.0	1.5	−4.0 to 9.0		
Sleep problems (2012; Jenkins)	9.3	4.1	4.0–24.0		
Sleep difficulties (2001; VEQ)	2.7	2.3	0.0–8.0		

Abbreviation: Vital Exhaustion Questionnaire.

**TABLE 3:** Bivariate correlation table of the study variables (*n* = 1064).

	1	2	3	4	5	6	7	8	9	10	11	12	13	14	15	16	17	18	19	20
1. Compass.	1.0																			
2. Age	.11^***^	1.0																		
3. Gender	.15^***^	.05^*^	1.0																	
4. Adult. income	.04^*^	.004	−.29^***^	1.0																
5. Adult. Occp. St.	.04^*^	−.03	−.20^***^	.49^***^	1.0															
6. Adult. Ed. Lev.	.09^***^	−.19^***^	.08^**^	.29^***^	.43^***^	1.0														
7. Parent. income	.05^**^	−.03	−.01	.17^***^	.22^***^	.21^***^	1.0													
8. Parent. Occp. Sta.	.05^**^	−.13^***^	−.01	.17^***^	.22^***^	.25^***^	.52^***^	1.0												
9. Parent. Ed. Lev.	.01	−.28^***^	−.05^**^	.14^***^	.21^***^	.26^***^	.48^***^	.70^***^	1.0											
10. BMI	−.01^***^	.12^***^	.07^***^	−.03	−.07^***^	−.12^***^	−.14^***^	−.17^***^	−.13^***^	1.0										
11. Phys. Act	.06^**^	−.04^*^	.08^***^	.12^***^	.09^*^	.13^***^	.09^***^	.07^***^	.05^**^	−.12^***^	1.0									
12. Alco. Cons.	−.16^***^	.03	−.32^***^	.06^**^	−.03	−.15^***^	−.02	.02	−.01	.12^***^	−.08^***^	1.0								
13. Smoking	−.05 ^*^	−.0002	−.05^**^	−.06^**^	−.10^***^	−.16^***^	−.07^***^	−.09^***^	−.010^***^	.03	−.14^***^	.21^***^	1.0							
14. Shift work	−.02	−.05^**^	−.02	−.18^***^	−.21^***^	−.13^***^	−.03	−.05^**^	−.05^**^	.03	−.01	−.01	.05^**^	1.0						
15. Empl. Sta.	.02	.05^**^	−.01	.24^***^	−.03	−.001	−.06^***^	−.08^***^	−.07^***^	−.05^**^	.08^***^	.02	.05^**^	−.13^***^	1.0					
16. Depre. Symp.	−.18^***^	.002	.08^***^	−.13^***^	−.004	−.03	−.05^**^	.02	.03	.11^***^	−.07^***^	−.0002	.01	.03	−.06^**^	1.0				
17. Sleep Durat.	.06^***^	−.05^**^	.16^***^	−.07^***^	−.002	.06^***^	.02	.01	.0002	−.06^***^	.002	.05^**^	−.07^***^	.01	−.03	−.09^***^	1.0			
18. Per. sleep Def.	−.08^***^	−.06^**^	.06^*^	−.004	.05^**^	−.01	−.02	.03	.06^**^	.02	−.02	−.03	.03	.03	−.004	.32^***^	−.50^***^	1.0		
19. Sleep Prob.	−.12^***^	.02	.08^***^	−.08^***^	−.03	−.06^***^	−.06^***^	.01	.03	.04^*^	−.06^**^	.04^*^	.004	.04	−.01	.51^***^	−.19^***^	.44^***^	1.0	
20. Sleep Diff.	−.15^***^	.03	.07^***^	−.13^***^	−.06^**^	−.05^**^	−.10^***^	−.04 ^*^	−.03	.10^***^	−.09^***^	.02	.02	.06^***^	−.02	.5^***^	−.14^***^	.42^**^	.75^***^	1.0

*Note*. Compass. = compassion, Adult. income = adulthood income, Adult. Occp. Sta. = adulthood occupational status, Adult. Ed. Lev. = adulthood educational level, Parent. income = parental income, Parent. Occp. Sta. = parental occupational status, Parent. Ed. Lev. = parental educational level, Phys. Act = physical activity, Alco. Cons. = alcohol consumption, Empl. Sta. = employment status, Depre. Symp. = depressive symptoms, Sleep Durat. = sleep duration, Per. Sleep. Def. = perceived sleep deficiency, Sleep Prob. = sleep problems, Sleep Diff. = sleep difficulties.

Abbreviation: BMI, body mass index.

* *p* < .05.

** *p* < .01.

*** *p* < .001.

### 3.1 Cross‐sectional associations between compassion and sleep indicators

Table 4 presents the results of multinomial logistic and linear regression when investigating the cross‐sectional association of compassion with sleep duration, perceived sleep deficiency, sleep problems (Jenkins), and sleep difficulties (VEQ), respectively. In Model 1 (adjusted for age, gender), high compassion was associated with lesser perceived sleep deficiency (*B* = −0.216, *p* < .01), fewer sleep problems (*B* = −0.979, *p* < .001), and sleep difficulties (*B* = −0.714, *p* < .001). In Model 2 (adjusted for age, gender, childhood and adulthood SEP, BMI, health behaviors, and working conditions), all the abovementioned associations remained significant. When further adjusted for depressive symptoms (Model 3), the association of compassion and sleep difficulties remained (*B* = −0.226, *p* < .05) but the relationship with perceived sleep deficiency and sleep problems disappeared. Compassion was not associated with sleep duration. The regression results of all the models with all the covariates can be found in Tables S1–S4.

**TABLE 4:** The results of linear and logistic regression analyses for the associations between compassion and sleep indicators in 2012.

	Model 1		Model 2		Model 3	
	*B*	CI 95%	*B*	CI 95%	*B*	CI 95%
Sleep duration						
6.5 h or less	−0.205	−.464; .054	−0.173	−.441; .096	−0.011	−.292; .269
7–8.5 h	(Ref.)	(Ref.)	(Ref.)	(Ref.)	(Ref.)	(Ref.)
9 h or more	0.049	−.049; .583	0.096	−.451; .643	0.307	−.257; .870
Sleep deficiency	−0.216^**^	−.374; −.058	−0.222^**^	−.382; −.062	−0.074	−.230; .081
Sleep problems	−0.979^***^	−1.401; −.556	−0.858^***^	−1.287; −.428	−0.219	−.600; .163
Sleep difficulties	−0.714^***^	−.966; −.461	−0.618^***^	−.872; −.364	−0.226^*^	−.447; −.004

*Note*: Estimates with 95% CI. Model 1: adjusted for age and gender; Model 2: adjusted for age, gender, SEP in childhood and adulthood, BMI, health behaviors, and working conditions; Model 3: adjusted for age, gender, SEP in childhood and adulthood, BMI, health behaviors, working conditions, and depressive symptoms.

Abbreviations: BMI, body mass index; CI, confidence intervals; SEP, socioeconomic position.

* *p* < .05.

** *p* < .01.

*** *p* < .001.

### 3.2 The longitudinal effect of compassion on the growth curve of sleep difficulties over 11 years

**FIGURE 1:**
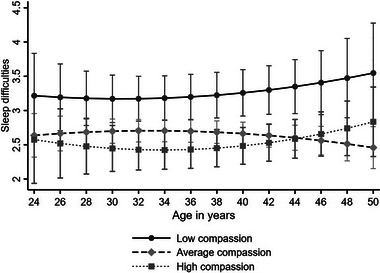
Model‐predicted values with 95% confidence intervals of sleep difficulties (Vital Exhaustion Questionnaire [VEQ] sleep items) over age separately for participants with low (−1 standard deviation [SD]), average, and high (+1 SD) levels of compassion (adjusted for age, gender, socioeconomic position [SEP] in childhood, and adulthood, body mass index [BMI], health behaviors, working conditions).

Next, we investigated the longitudinal effect of compassion on the trajectory of sleep difficulties over the 11‐year follow‐up. The results of the multilevel models are presented in Table 5. High compassion predicted fewer sleep difficulties when adjusted for age and gender (Model 1, *B* = −0.520, *p* < .05) and when controlling for SEP factors in childhood and adulthood, BMI, health behaviors, and working conditions (Model 2, *B* = −0.448, *p* < .05). The age‐interaction of compassion was nonsignificant, indicating that the effect of compassion on sleep difficulties was evident over age (between the ages of 24–50 years). Model 2 is depicted in Figure 1. When further adjusting for depressive symptoms (Model 3), the effect of compassion on sleep difficulties disappeared. The results of all the models with all the covariates can be found in Table S5.

**TABLE 5:** Results of the growth curve model with a longitudinal design.

	Model 1		Model 2		Model 3	
	*B*	CI 95%	*B*	CI 95%	*B*	CI 95%
Fixed effects						
Intercept	4.253^***^	2.768; 5.738	4.492^***^	2.495; 6.488	2.892^**^	.971; 4.812
Age	−0.026	−.245; .192	−0.019	−.238; .201	−0.011	−.233; .210
Compassion × age	0.009	−.050; .067	0.007	−.052; .066	0.005	−.054; .065
Compassion × age × age	−0.001	−.003; .002	−0.001	−.003; .002	−0.001	−.003; .002
Compassion	−0.520^*^	−.919; −.119	−0.448^*^	−.853; .043	−0.187	−.599; .225
Random effects						
Variance of intercept	1.277^*^	.921; 1.771	1.299^*^	1.023; 1.648	1.067^*^	.874; 1.302
Residual variance	1.688^*^	1.608; 1.771	1.689^*^	1.622; 1.759	1.695^*^	1.640; 1.752

*Note*: Estimates (B) with 95% CI of compassion and age, when predicting sleep difficulties VEQ. Model 1: adjusted for age and gender; Model 2: adjusted for age, gender, SEP in childhood and adulthood, BMI, health behaviors, and working conditions; Model 3: adjusted for age, gender, SEP in childhood and adulthood, BMI, health behaviors, working conditions, and depressive symptoms.

Abbreviations: BMI, body mass index; CI, confidence intervals; SEP, socioeconomic position; VEQ, Vital Exhaustion Questionnaire.

* *p* < .05.

** *p* < .01.

*** *p* < .001.

### 3.3 The temporal relationship between dispositional compassion and sleep difficulties

**FIGURE 2:**
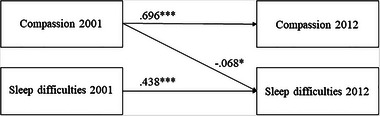
An illustration of the predictive pathway from compassion to sleep difficulties Vital Exhaustion Questionnaire [(VEQ]). The covariates (age, gender, and socioeconomic position [SEP] in childhood and adulthood) and covariances between compassion and sleep difficulties in each measurement year were omitted from the figure for clarity. ^*^ *p* < .05; ^***^ *p* < .001.

The predictive relationships between compassion and sleep difficulties were assessed using cross‐lagged panel models (Table 6). All the models had a good statistical fit (RMSEA ≤ .038, CFI ≥ 0.974). The *χ* ^2^ difference test and the CFI and RMSEA values, however, showed that Model 2 (a predictive pathway from compassion to sleep difficulties) was better than Model 1 (no predictive pathways) (*p* < .05 in the *χ* ^2^ difference test) or Model 3 (a predictive pathway from sleep difficulties to compassion). Moreover, Model 4 (bidirectional predictive pathways) was not better than Model 2 in the *χ* ^2^ difference test (*p* > .05). Taken together, the predictive pathways seemed to proceed more likely from high compassion to fewer sleep difficulties than in the opposite direction (see Figure 2). All the models were adjusted for age, gender, and SEP in childhood and adulthood.

**TABLE 6:** The goodness‐of‐fit statistics for the longitudinal models on the predictive relationships of compassion with sleep difficulties (adjusted for age, gender, childhood, and adulthood socioeconomic position (SEP).

							Model comparisons		
	*χ* ^2^ value	*df*	*p*	RMSEA	CFI	BIC	*χ* ^2^ difference test	*df*	*p*
Model 1	40.855	18	.002	.038	0.974	31,430.076			
Model 2	36.256	17	.004	.036	0.978	31,432.266	(2 vs. 1) = 4.60	1	.032
Model 3	39.181	17	.002	.038	0.975	31,435.191	(3 vs. 1) = 1.67	1	.196
Model 4	34.633	16	.004	.036	0.979	31,437.432	(4 vs. 1) = 6.22	2	.045
							(4 vs. 2) = 1.62	2	.203

*Note*: Model 1: no cross‐lagged predictive paths; Model 2: predictive paths from compassion to sleep difficulties; Model 3: predictive paths from sleep difficulties to compassion; Model 4: bidirectional predictive paths.

Abbreviations: BIC, Bayesian information criterion; CFI, comparative fit index; RMSEA, root mean square error of approximation.

## 4 DISCUSSION

The current study was the first to examine the association between dispositional compassion for others and sleep. Using a comparatively large population‐based sample, we found that high compassion was cross‐sectionally associated with better sleep, including lesser perceived sleep deficiency and fewer sleep difficulties. Compassion was not associated with sleep duration (i.e., the likelihood of being a short or long sleeper). High compassion also longitudinally predicted fewer sleep difficulties over the 11‐year follow‐up. The results remained after controlling for age, gender, SEP in childhood and adulthood, health behaviors, BMI, and working conditions. Lastly, the results showed that the predictive pathway is more likely to proceed from high compassion to lower sleep difficulties than in the opposite direction (i.e., from low sleep difficulties to higher compassion).

We also found that the effect of compassion on most sleep indicators (with the exception of sleep difficulties in the cross‐sectional analysis) disappeared after controlling for depressive symptoms, possibly implying that depressive symptoms mediate the pathway from high compassion to fewer sleep issues. Interestingly, in previous studies high compassion has predicted fewer depressive symptoms over a 15‐year follow‐up, rather than low depressive symptoms predicting higher compassion (Saarinen et al., 2019). Moreover, several compassion intervention studies have found that compassion is associated with reduced depressive symptoms (Kirby et al., 2017). Lower depressive symptoms, in turn, are associated with, for example, decreased sleep onset latency and less frequent sleep disruptions (Pandi‐Perumal et al., 2020). Additionally, while compassion is other‐focused (Goetz et al., 2010), depression typically includes a maladaptive self‐focus such as rumination on past failures (Strauman, 2002). A negative self‐focus, in turn, can hinder relaxation in the nighttime and promote wakefulness (Butz & Stahlberg, 2018; Hu et al., 2018). Taken together, it appears plausible that the protective effect of compassion against sleep difficulties may be mediated by depressive symptoms.

The association from high compassion to fewer sleep difficulties may also be explained by stress regulation. Research has shown, for instance, that high compassion is associated with lower self‐reported and physiologically measured levels of stress (Abelson et al., 2014; Engert et al., 2017; Saarinen et al., 2021), including the stronger activity of the vagus nerve of the parasympathetic nervous system (Porges, 2001; Stellar & Keltner, 2017). High compassion is also found to be associated with more adaptive coping styles in stressful situations, for instance, greater acceptance in the face of adversity (Jazaieri et al., 2018). Experienced stress, in turn, is a well‐known risk factor for sleep difficulties, such as sleep discontinuity, sleep deficiency, and lesser amounts of deep sleep (Åkerstedt, 2006).

Another possible stress‐related mechanism between compassion and sleep is social support. There is evidence that high compassion is cross‐sectionally and longitudinally associated with higher perceived social connectedness and social support (Cosley et al., 2010; Gilbert, 2017; Saarinen, Keltikangas‐Järvinen, Pulkki‐Råback, et al., 2020) that, in turn, is linked with better sleep (de Grey et al., 2018; Magnusson Hanson et al., 2011). Social support is a well‐established stress reliever as social support has been associated with increased emotional, instrumental, informational and appraisal support, which reduces negative affect and increases positive affect, perceived control, and competence, among others (Langford et al., 1997; Taylor, 2012). Thus, there may be both physiological and psychosocial mechanisms that explain the relationship between compassion and sleep.

Some limitations are necessary to be taken into consideration. First, sleep indicators were measured with self‐report questionnaires, subjective to possible bias. Self‐reported and physiological sleep measurements are also found to result in somewhat discrepant results (Fernandez‐Mendoza et al., 2011). There is evidence, however, that subjective sleep measurements predict sleep‐related inflammatory biomarkers over a 5‐year follow‐up (Prather et al., 2013) and cardiovascular risk over a 15‐year follow‐up (Hoevenaar‐Blom et al., 2011), implying that self‐reports on sleep may still result in convergent findings over a long‐term follow‐up. Second, compassion was measured twice and sleep difficulty items thrice during the follow‐up of a total of 11 years, meaning in the growth curve analysis, we were able to include compassion only from the first measurement point. In addition, the short‐term effects of compassion on sleep difficulties remain unknown for now, although, the long 11‐year follow‐up was a considerable strength of the study. Lastly, although sleep difficulties were measured three times over 11 years, the scale does not originally measure only sleep.

The current study had also other several strengths. We had a comparatively large and population‐based sample on compassion and sleep indicators. Second, we used a variety of sleep indicators (i.e., sleep duration, perceived sleep deficiency, difficulties with sleep) that provided quite a comprehensive picture of the quality and quantity of sleep. Thus, the addition of the several variables offers not only robustness but also an opportunity to see if the results replicate with different measurements. Third, the longitudinal data on sleep difficulties also allowed the examination of longitudinal and temporal associations, in addition to cross‐sectional ones with other sleep measurements. Lastly, the YFS data provided good possibilities to take into consideration several potential confounders, including the SEP in childhood and adulthood, BMI, health behaviors (alcohol use, smoking, and physical activity), working conditions (employment status and shift work), and depressive symptoms.

Currently, sleep difficulties are increasingly prevalent (Kronholm et al., 2016; Léger et al., 2008) with many sleep difficulties remaining undertreated (Chattu et al., 2019) At the same time, the efficiency of sleep medications has been noted to be limited, partly because of side effects (Pagel et al., 2018). Thus, there is an increasing need for novel interventions to reduce sleep difficulties. Our findings provide the first pieces of evidence that compassion for others could buffer against sleep difficulties. Moreover, we found that compassion was associated with less perceived sleep deficiency, sleep problems, and sleep difficulties, even after accounting for work conditions, one of the most frequently reported causes of sleep issues factors (Åkerstedt et al., 2002; Törnroos et al., 2017) as well as several other factors related to sleep. As compassion has been found to predict a variety of well‐being factors (rather than high well‐being predicting higher compassion) (Saarinen, Keltikangas‐Järvinen, Pulkki‐Råback, et al., 2020), compassion‐focused interventions could also be effective means to reduce sleep difficulties. As cost‐effective compassion interventions require comparatively brief initial training, increase compassion in a few months’ time, and are flexible in the administration, time, and setting wise, they might be relatively accessible to various groups (Kirby, 2017). The effectiveness of compassion interventions as a preventive tool or as a light intervention against sleep difficulties, however, requires further research.

## AUTHOR CONTRIBUTIONS

Iina Tolonen analyzed the data and wrote the first draft. Iina Tolonen, Aino Saarinen, and Mirka Hintsanen collaborated on the design, initial interpretations of the results, and compiling the manuscript. Aino Saarinen supervised the data analyses. All authors discussed the design, the results, and commented on the manuscript. Authors read and agreed upon the final version of the manuscript.

## CONFLICT OF INTEREST STATEMENT

The authors declare that there are no conflicts of interest that could be perceived as prejudicing the impartiality of the research reported.

### CONSENT TO PARTICIPATE

All the participants or their parents (if participants aged <18 years) provided informed consent before participation.

### CONSENT FOR PUBLICATION

The participants have consented to the submission of their data to the journal.

### PEER REVIEW

The peer review history for this article is available at https://publons.com/publon/10.1002/brb3.3165.

## Supporting information


**Table S1** The results of regression analyses of compassion and risk‐factors predicting sleep duration: short and long sleepers (intermediate sleepers as a reference group).
**Table S2** The results of regression analyses of compassion and risk‐factors predicting sleep deficiency.
**Table S3** The results of regression analyses of compassion and risk‐factors predicting sleep problems.
**Table S4** The results of regression analyses of compassion and risk‐factors predicting sleep difficulties.
**Table S5** Results of the growth curve model with a longitudinal design. Estimates (B) with 95% confidence intervals (CI) of compassion and age, when predicting sleep difficulties (VEQ).

## Data Availability

As the Young Finns Study is an ongoing, prospective study, the datasets are not readily available. The datasets are not anonymized, and General Data Protection Regulation prevents the public sharing of the data. However, pseudonymized datasets can be requested. In this case, a data sharing agreement is done between the parties. Requests for these datasets should be directed to Liisa Keltikangas‐Järvinen (liisa.keltikangas-jarvinen@helsinki.fi) for compassion data and to Katri Räikkönen (katri.raikkonen@helsinki.fi) or Niklas Ravaja (niklas.ravaja@helsinki.fi) for other psychological data.
