# Supplementary material for: High compassion predicts fewer sleep difficulties: A general population study with an 11‐year follow‐up
Source: Brain Behav. 2023 Aug 22;13(10):e3165. doi: 10.1002/brb3.3165 (PMC10570475; doi:10.1002/brb3.3165)
Supplement: Supplementary file 1 — Table S1 The results of regression analyses of compassion and risk‐factors predicting sleep duration: short and long sleepers (intermediate sleepers as a reference group). Table S2 The results of regression analyses of compassion and risk‐factors predicting sleep deficiency. Table S3 The results of regression analyses of compassion and risk‐factors predicting sleep problems. Table S4 The results of regression analyses of compassion and risk‐factors predicting sleep difficulties. Table S5 Results of the growth curve model with a longitudinal design. Estimates (B) with 95% confidence intervals (CI) of compassion and age, when predicting sleep difficulties (VEQ). [file BRB3-13-e3165-s001.docx]

**Supplementary Table 1.**

*The results of regression analyses of compassion and risk-factors predicting sleep duration: short and long sleepers (intermediate sleepers as a reference group)*

|  | Model 1 | | | | Model 2 | | | | Model 3 | | | |
| --- | --- | --- | --- | --- | --- | --- | --- | --- | --- | --- | --- | --- |
|  | Pseudo *R*^2^ = 0.023 | | | | Pseudo *R*^2^ = 0.049 | | | | Pseudo *R*^2^ = 0.086 | | | |
|  | Short Sleepers | | Long Sleepers | | Short Sleepers | | Long Sleepers | | Short Sleepers | | Long Sleepers | |
|  | B | CI 95% | B | CI 95% | B | CI 95% | B | CI 95% | B | CI 95% | B | CI 95% |
| Age | 0.050** | 0.018;0.081 | -0.023 | -0.085;0.038 | 0.046** | 0.012;0.081 | -0.032 | -0.099; 0.036 | 0.046* | 0.011; 0.080 | -0.035 | -0.104;0.033 |
| Gender | -0.576*** | -0.883;0.269 | 0.613 | -0.072;1.297 | -0.557** | -0.910;-0.204 | 0.604 | -0.155; 1.364 | -0.681*** | -1.046;-0.316 | 0.463 | -0.300;1.227 |
| Par.Income |  |  |  |  | -0.067 | -0.161; 0.028 | 0.078 | -0.120; 0.275 | -0.052 | -0.148; 0.044 | 0.097 | -0.100;0.294 |
| Par.Ed.Lev. |  |  |  |  |  |  |  |  |  |  |  |  |
| Comp.Sch. |  |  |  |  | -0.162 | -0.577; 0.254 | 0.387 | -0.447; 1.221 | -0.178 | -0.602; 0.246 | 0.376 | -0.468;1.220 |
| High/Vo.Sch. |  |  |  |  | (Ref.) | (Ref.) | (Ref.) | (Ref.) | (Ref.) | (Ref.) | (Ref.) | (Ref.) |
| College/Univ. |  |  |  |  | -0.206 | -0.729; 0.318 | -0.638 | -1.681; 0.404 | -0.335 | -0.876; 0.205 | -0.758 | -1.812;0.296 |
| Par.Occ.St |  |  |  |  |  |  |  |  |  |  |  |  |
| Manual |  |  |  |  | -0.096 | -0.498; 0.305 | -0.398 | -1.223; 0.427 | -0.057 | -0.468; 0.354 | -0.381 | -1.218;0.455 |
| Lo.non-man |  |  |  |  | (Ref.) | (Ref.) | (Ref.) | (Ref.) | (Ref.) | (Ref.) | (Ref.) | (Ref.) |
| Up.non-man |  |  |  |  | 0.067 | -0.515; 0.648 | 0.083 | -1.048; 1.214 | 0.072 | -0.530; 0.675 | 0.067 | -1.084;1.217 |
| Adult.Income |  |  |  |  | 0.015 | -0.055; 0.085 | -0.030 | -0.172; 0.111 | 0.041 | -0.032; 0.113 | -0.001 | -0.144;0.142 |
| Adult.Ed.Lev. |  |  |  |  |  |  |  |  |  |  |  |  |
| Compre.Sch. |  |  |  |  | -0.210 | -0.901; 0.481 | 0.131 | -1.397; 1.658 | -0.215 | -0.920; 0.491 | 0.122 | -1.422;1.665 |
| High/Voc.Sch. |  |  |  |  | (Ref.) | (Ref.) | (Ref.) | (Ref.) | (Ref.) | (Ref.) | (Ref.) | (Ref.) |
| Colleg/Univ. |  |  |  |  | -0.329 | -0.723; 0.064 | 0.617 | -0.116; 1.350 | -0.319 | -0.723; 0.084 | 0.652 | -0.088;1.391 |
| Adult.Occ.St. |  |  |  |  |  |  |  |  |  |  |  |  |
| Manual |  |  |  |  | 0.297 | -0.146; 0.741 | -0.081 | -0.881; 0.720 | 0.358 | -0.097; 0.812 | -0.017 | -0.829;0.796 |
| Lo.non-man |  |  |  |  | (Ref.) | (Ref.) | (Ref.) | (Ref.) | (Ref.) | (Ref.) | (Ref.) | (Ref.) |
| Up.non-man |  |  |  |  | 0.529 | 0.006; 1.053 | -0.386 | -1.398; 0.626 | 0.445 | -0.092; 0.982 | -0.483 | -1.509;0.544 |
| BMI |  |  |  |  | 0.050** | 0.019; 0.081 | 0.029 | -0.030; 0.089 | 0.040 | 0.008; 0.072 | 0.018 | -0.042;0.078 |
| Phys.Activity |  |  |  |  | 0.089 | -0.002; 0.179 | -0.017 | -0.200; 0.165 | 0.111* | 0.018; 0.203 | -0.001 | -0.185;0.182 |
| Alc.Consump. |  |  |  |  | -0.021 | -0.135; 0.092 | 0.135 | -0.088; 0.358 | -0.012 | -0.128; 0.104 | 0.136 | -0.090;0.361 |
| Smoking |  |  |  |  | 0.438 | -0.025; 0.901 | 0.204 | -0.754; 1.162 | 0.451 | -0.023; 0.925 | 0.281 | -0.684;1.246 |
| Shiftwork |  |  |  |  | 0.106 | -0.244; 0.456 | 0.134 | -0.557; 0.826 | 0.069 | -0.291; 0.428 | 0.098 | -0.598;0.795 |
| Employment St. |  |  |  |  | 0.059 | -0.554; 0.672 | -0.410 | -1.399; 0.579 | 0.094 | -0.542; 0.729 | -0.388 | 1.395; 0.619 |
| Dep.Sympt. |  |  |  |  |  |  |  |  | 0.088*** | 0.062; 0.115 | 0.090*** | 0.046; 0.134 |
| Compassion | -0.205 | -0.464; 0.054 | 0.049 | -0.485;0.583 | -0.173 | -0.441; 0.096 | 0.096 | -0.451; 0.643 | -0.011 | -0.292; 0.269 | 0.307 | -0.257;0.870 |
| **Model 1**: Adjusted for age and gender.  **Model 2:** Adjusted for age, gender, SEP in childhood (parental income, parental educational level, parental occupational status) and adulthood (adulthood income, educational level, occupational status), BMI, health behaviors (physical activity, alcohol consumption, smoking), and working conditions (shift work, employment status).  **Model 3**: Adjusted for age, gender, SEP in childhood (parental income, parental educational level, parental occupational status) and adulthood (adulthood income, educational level, occupational status), BMI, health behaviors (physical activity, alcohol consumption, smoking), and working conditions (shift work, employment status), and depressive symptoms.  **p* < 0.05. ***p* < 0.01. ****p* < 0.001 | | | | | | | | | | | | |

**Supplementary Table 2.**

*The results of regression analyses of compassion and risk-factors predicting sleep deficiency*

|  | Model 1 | | Model 2 | | Model 3 | |
| --- | --- | --- | --- | --- | --- | --- |
|  | Adjusted *R*^2^ = 0.011 | | Adjusted *R*^2^ = 0.021 | | Adjusted *R*^2^ = 0.110 | |
|  | B | CI 95% | B | CI 95% | B | CI 95% |
| Age | -0.016 | -0.034; 0.003 | -0.009 | -0.029; 0.011 | -0.074 | -0.230; 0.081 |
| Gender | 0.217* | 0.031; 0.402 | 0.264* | 0.056; 0.472 | 0.179 | -0.020; 0.378 |
| Parental Income |  |  | -0.050 | -0.106; 0.007 | -0.034 | -0.088; 0.020 |
| Parental Edu. Lev. |  |  |  |  |  |  |
| Compre. School |  |  | -0.298* | -0.547; -0.048 | -0.301* | -0.539; -0.063 |
| High/Voc. School |  |  | (Ref.) | (Ref.) | (Ref.) | (Ref.) |
| College/University |  |  | -0.069 | -0.364; 0.226 | -0.152 | -0.433; 0.130 |
| Parental Occup. St. |  |  |  |  |  |  |
| Manual |  |  | 0.112 | -0.130; 0.353 | 0.148 | -0.082; 0.379 |
| Lower non-manual |  |  | (Ref.) | (Ref.) | (Ref.) | (Ref.) |
| Upper non-manual |  |  | 0.304 | -0.021; 0.628 | 0.293 | -0.017; 0.603 |
| Adult. Income |  |  | -0.003 | -0.045; 0.038 | 0.019 | -0.021; 0.058 |
| Adult. Edu Lev. |  |  |  |  |  |  |
| Compre. School |  |  | 0.091 | -0.330; 0.511 | 0.104 | -0.297; 0.505 |
| High/Voc. School |  |  | (Ref.) | (Ref.) | (Ref.) | (Ref.) |
| College/University |  |  | -0.166 | -0.391; 0.059 | -0.147 | -0.361; 0.067 |
| Adult Occup. St. |  |  |  |  |  |  |
| Manual |  |  | 0.091 | -0.157; 0.339 | 0.118 | -0.119; 0.355 |
| Lower non-manual |  |  | (Ref.) | (Ref.) | (Ref.) | (Ref.) |
| Upper non-manual |  |  | 0.470** | 0.172; 0.767 | 0.392** | 0.108; 0.676 |
| BMI |  |  | 0.006 | -0.013; 0.025 | -0.004 | -0.022; 0.015 |
| Physical Activity |  |  | -0.007 | -0.060; 0.047 | 0.006 | -0.045; 0.056 |
| Alcohol consump. |  |  | -0.033 | -0.101; 0.036 | -0.028 | -0.093; 0.037 |
| Smoking |  |  | 0.152 | -0.144; 0.447 | 0.150 | -0.132; .431 |
| Shiftwork |  |  | 0.119 | -0.090; 0.327 | 0.098 | -0.101; 0.297 |
| Employment status |  |  | 0.024 | -0.321; 0.368 | 0.044 | -0.285; 0.372 |
| Depressive sympt. |  |  |  |  | 0.080*** | 0.064; 0.095 |
| Compassion | -0.216* | -0.374; -0.058 | -0.222** | 0.382; 0.062 | -0.074 | -0.230; 0.081 |
| **Model 1:** Adjusted for age and gender.  **Model 2:** Adjusted for age, gender, SEP in childhood (parental income, parental educational level, parental occupational status) and adulthood (adulthood income, educational level, occupational status), BMI, health behaviors (physical activity, alcohol consumption, smoking), and working conditions (shift work, employment status).  **Model 3**: Adjusted for age, gender, SEP in childhood (parental income, parental educational level, parental occupational status) and adulthood (adulthood income, educational level, occupational status), BMI, health behaviors (physical activity, alcohol consumption, smoking), and working conditions (shift work, employment status), and depressive symptoms  **p* < 0.05. ***p* < 0.01. ****p* < 0.001 | | | | | | |

**Supplementary Table 3.**

*The results of regression analyses of compassion and risk-factors predicting sleep problems*

|  | Model 1 | | Model 2 | | Model 3 | |
| --- | --- | --- | --- | --- | --- | --- |
|  | Adjusted *R*^2^ = 0.022 | | Adjusted *R*^2^ = 0.030 | | Adjusted *R*^2^ = 0.261 | |
|  | B | CI 95% | B | CI 95% | B | CI 95% |
| Age | 0.021 | -0.028; 0.070 | 0.025 | -0.028; 0.078 | -0.219 | -0.600; 0.163 |
| Gender | 0.804** | 0.306; 1.302 | 0.967** | 0.409; 1.525 | 0.600* | 0.112; 1.088 |
| Parental Income |  |  | -0.191* | -0.343; -0.039 | -0.124 | -0.257; 0.009 |
| Parental Edu. Lev. |  |  |  |  |  |  |
| Compre.School |  |  | -0.703* | -1.373; -0.033 | -0.717* | -1.301; -0.133 |
| High/Voc.School |  |  | (Ref.) | (Ref.) | (Ref.) | (Ref.) |
| College/University |  |  | 0.053 | -0.738; 0.843 | -0.305 | -0.996; 0.385 |
| Parental Occup. St. |  |  |  |  |  |  |
| Manual |  |  | 0.003 | -0.645; 0.650 | 0.161 | -0.404; 0.726 |
| Lower non-manual |  |  | (Ref.) | (Ref.) | (Ref.) | (Ref.) |
| Upper non-manual |  |  | 0.575 | -0.296; 1.446 | 0.530 | -0.230; 1.290 |
| Adult. Income |  |  | -0.055 | -0.166; 0.056 | 0.039 | -0.058; 0.136 |
| Adult. Edu Lev. |  |  |  |  |  |  |
| Compr. School |  |  | -0.201 | -1.329; 0.927 | -0.144 | -1.128; 0.840 |
| High/Voc. School |  |  | (Ref.) | (Ref.) | (Ref.) | (Ref.) |
| College/University |  |  | -0.522 | -1.125; 0.081 | -0.438 | -0.965; 0.088 |
| Adult Occup. St. |  |  |  |  |  |  |
| Manual |  |  | -0.407 | -1.073; 0.259 | -0.291 | -0.872; 0.291 |
| Lower non-manual |  |  | (Ref.) | (Ref.) | (Ref.) | (Ref.) |
| Upper non-manual |  |  | 0.004 | -0.793; 0.801 | -0.332 | -1.028; 0.365 |
| BMI |  |  | 0.017 | -0.034; 0.069 | -0.024 | -0.069; 0.021 |
| Physical Activity |  |  | -0.087 | -0.229; 0.056 | -0.034 | -0.159; 0.090 |
| Alcohol consump. |  |  | 0.143 | -0.040; 0.326 | 0.162 | 0.003; 0.322 |
| Smoking |  |  | -0.200 | -0.991; 0.592 | -0.209 | -0.900; 0.482 |
| Shiftwork |  |  | 0.312 | -0.248; 0.871 | 0.224 | -0.264; 0.713 |
| Employment status |  |  | 0.238 | -0.686; 1.161 | 0.324 | -0.482; 1.130 |
| Depressive sympt. |  |  |  |  | 0.343*** | 0.306; 0.381 |
| Compassion | -0.979*** | -1.401; -0.558 | -0.858*** | -1.287; -0.428 | -0.219 | -0.600; 0.163 |
| **Model 1:** Adjusted for age and gender.  **Model 2:** Adjusted for age, gender, SEP in childhood (parental income, parental educational level, parental occupational status) and adulthood (adulthood income, educational level, occupational status), BMI, health behaviors (physical activity, alcohol consumption, smoking), and working conditions (shift work, employment status).  **Model 3**: Adjusted for age, gender, SEP in childhood (parental income, parental educational level, parental occupational status) and adulthood (adulthood income, educational level, occupational status), BMI, health behaviors (physical activity, alcohol consumption, smoking), and working conditions (shift work, employment status), and depressive symptoms  **p* < 0.05. ***p* < 0.01. ****p* < 0.001 | | | | | | |

**Supplementary Table 4.**

*The results of regression analyses of compassion and risk-factors predicting sleep difficulties*

|  | Model 1 | | Model 2 | | Model 3 | |
| --- | --- | --- | --- | --- | --- | --- |
|  | Adjusted *R*^2^ = 0.031 | | Adjusted *R*^2^ = 0.054 | | Adjusted *R*^2^ = 0.303 | |
|  | B | CI 95% | B | CI 95% | B | CI 95% |
| Age | 0.019 | -0.010; 0.049 | 0.014 | -0.0170; 0.046 | 0.007 | -0.020; 0.034 |
| Gender | 0.470** | 0.173; 0.766 | 0.437** | 0.108; 0.766 | 0.210 | -0.074; 0.493 |
| Parental Income |  |  | -0.114* | -0.204; -.024 | -0.072 | -0.149; 0.005 |
| Parental Edu. Lev. |  |  |  |  |  |  |
| Compre. School |  |  | 0.054 | -0.341; 0.449 | 0.050 | -0.289; 0.389 |
| High/Voc. School |  |  | (Ref.) | (Ref.) | (Ref.) | (Ref.) |
| College/University |  |  | 0.280 | -0.189; 0.749 | 0.057 | -0.346; 0.460 |
| Parental Occup.St |  |  |  |  |  |  |
| Manual |  |  | 0.049 | -0.333; 0.431 | 0.152 | -0.176; 0.480 |
| Lower non-manual |  |  | (Ref.) | (Ref.) | (Ref.) | (Ref.) |
| Upper non-manual |  |  | 0.210 | -0.306; 0.726 | 0.184 | -0.259; 0.627 |
| Adult. Income |  |  | -0.083* | -0.148; -0.017 | -0.024 | -0.081; 0.032 |
| Adult. Edu. Lev. |  |  |  |  |  |  |
| Compre.School |  |  | -0.649 | -1.314; 0.015 | -0.615* | -1.185; -0.044 |
| High/Vocat.School |  |  | (Ref.) | (Ref.) | (Ref.) | (Ref.) |
| College/University |  |  | -0.108 | -0.464; 0.248 | -0.061 | -0.366; 0.245 |
| Adult. Occup.Stat. |  |  |  |  |  |  |
| Manual |  |  | -0.294 | -0.687; 0.099 | -0.218 | -0.556; 0.119 |
| Lower non-manual |  |  | (Ref.) | (Ref.) | (Ref.) | (Ref.) |
| Upper non-manual |  |  | -0.109 | -0.579; 0.362 | -0.311 | -0.716; 0.093 |
| BMI |  |  | 0.036* | 0.006; 0.066 | 0.010 | -0.016; 0.036 |
| Physical Activity |  |  | -0.095* | -0.179; -0.011 | -0.062 | -0.134; 0.011 |
| Alcohol Consump. |  |  | 0.030 | -0.077; 0.138 | 0.044 | -0.048; 0.137 |
| Smoking |  |  | 0.0001 | -0.468; 0.469 | -0.018 | -0.421; 0.384 |
| Shiftwork |  |  | 0..247 | -0.083; 0.577 | -0.018 | -0.095; 0.472 |
| Employment Status |  |  | 0.214 | -0.330; 0.758 | 0.189 | -0.206; 0.728 |
| Depressive Sympt. |  |  |  |  | 0.212*** | 0.191; 0.234 |
| Compassion | -0.714*** | -0.966; -0.461 | -0.618*** | -0.872; -0.364 | -0.226* | -0.447; -0.003 |
| **Model 1**: Adjusted for age and gender.  **Model 2:** Adjusted for age, gender, SEP in childhood (parental income, parental educational level, parental occupational status) and adulthood (adulthood income, educational level, occupational status), BMI, health behaviors (physical activity, alcohol consumption, smoking), and working conditions (shift work, employment status).  **Model 3**: Adjusted for age, gender, SEP in childhood (parental income, parental educational level, parental occupational status) and adulthood (adulthood income, educational level, occupational status), BMI, health behaviors (physical activity, alcohol consumption, smoking), and working conditions (shift work, employment status), and depressive symptoms.  **p* < 0.05. ***p* < 0.01. ****p* < 0.001 | | | | | | |

**Supplementary Table 5.**

*Results of the growth curve model with a longitudinal design. Estimates (B) with 95% confidence intervals (CI) of compassion and age, when predicting sleep difficulties (VEQ).*

|  | Model 1 | | Model 2 | | Model 3 | |
| --- | --- | --- | --- | --- | --- | --- |
|  | B | CI 95% | B | CI 95% | B | CI 95% |
| **Fixed effects** |  |  |  |  |  |  |
| Intercept | 4.253*** | 2.768; 5.738 | 5.105*** | 3.146; 7.064 | 3.375*** | 1.480; 5.269 |
| Age | -0.026 | -0.245; 0.192 | -0.019 | -0.238; 0.201 | -0.011 | -0.233; 0.210 |
| Gender | 0.597*** | 0.341; 0.853 | 0.622*** | 0.340; 0.904 | 0.460*** | 0.212; 0.709 |
| Parental Income |  |  | -0.051 | -0.128; 0.026 | -0.035 | -0.103; 0.032 |
| Parental Edu. Lev. |  |  |  |  |  |  |
| Compre. School |  |  | -0.169 | -0.504; 0.167 | -0.167 | -0.459; 0.125 |
| High/Voc. School |  |  | (Ref.) | (Ref.) | (Ref.) | (Ref.) |
| College/University |  |  | 0.162 | -0.232; 0.556 | -0.045 | -0.396; 0.306 |
| Parental Occup.Sta. |  |  |  |  |  |  |
| Manual |  |  | 0.169 | -0.156; 0.494 | 0.243 | -0.041; 0.527 |
| Lower non-manual |  |  | (Ref.) | (Ref.) | (Ref.) | (Ref.) |
| Upper non-manual |  |  | 0.109 | -0.325; 0.544 | 0.140 | -0.246; 0.526 |
| Adult. Income |  |  | -0.039 | -0.096; 0.018 | -0.005 | -0.055; 0.046 |
| Adult. Edu. Lev. |  |  |  |  |  |  |
| Compre. School |  |  | -0.547 | -1.141; 0.047 | -0.523* | -1.037; -0.009 |
| High/Voc. School |  |  | (Ref.) | (Ref.) | (Ref.) | (Ref.) |
| College/University |  |  | -0.097 | -0.394; 0.199 | -0.016 | -0.276; 0.245 |
| Adult. Occup. Sta. |  |  |  |  |  |  |
| Manual |  |  | -0.067 | -0.402; 0.268 | -0.036 | -0.327; 0.256 |
| Lower non-manual |  |  | (Ref.) | (Ref.) | (Ref.) | (Ref.) |
| Upper non-manual |  |  | 0.051 | -0.349; 0.451 | -0.129 | -0.480; 0.223 |
| BMI |  |  | 0.018 | -0.008; 0.044 | 0.001 | -0.022; 0.025 |
| Physical activity |  |  | -0.104** | -0.174; -0.033 | -0.083** | -0.145; -0.021 |
| Alcohol consump. |  |  | 0.024 | -0.071; 0.119 | 0.030 | -0.052; 0.112 |
| Smoking |  |  | 0.121 | -0.282; 0.525 | 0.106 | -0.248; 0.460 |
| Shiftwork |  |  | -0.059 | -0.516; 0.399 | -0.051 | -0.298; 0.195 |
| Employment Status |  |  | -0.037 | -0.318; 0.245 | -0.005 | -0.408; 0.397 |
| Depressive sympt. |  |  |  |  | 0.166*** | 0.147; 0.185 |
| Compassion*Age | 0.008 | -0.050; 0.067 | 0.007 | -0.052; 0.066 | 0.005 | -0.054; 0.065 |
| Compassion*Age*Age | -0.001 | -0.003; 0.002 | -0.001 | -0.003; 0.002 | -0.001 | -0.003; 0.002 |
| Compassion | -0.519* | -0.919; -0.119 | -0.448* | -0.853; -0.043 | -0.187 | -0.599; 0.225 |
|  |  |  |  |  |  |  |
| **Random effects** |  |  |  |  |  |  |
| Variance of intercept | 1.277* | 0.921; 1.771 | 1.299* | 1.014; 1.663 | 1.330* | 1.049; 1.686 |
| Residual variance | 1.688* | 1.608; 1.771 | 1.689* | 1.621; 1.760 | 1.668* | 1.622; 1.757 |
| **Model 1:** Adjusted for age and gender.  **Model 2:** Adjusted for age, gender, SEP in childhood (parental income, parental educational level, parental occupational status) and adulthood (adulthood income, educational level, occupational status), BMI, health behaviors (physical activity, alcohol consumption, smoking), and working conditions (shift work, employment status).  **Model 3**: Adjusted for age, gender, SEP in childhood (parental income, parental educational level, parental occupational status) and adulthood (adulthood income, educational level, occupational status), BMI, health behaviors (physical activity, alcohol consumption, smoking), and working conditions (shift work, employment status), and depressive symptoms  **p* < 0.05. ***p* < 0.01. ****p* < 0.001 | | | | | | |
